# NetMODE: Network Motif Detection without Nauty

**DOI:** 10.1371/journal.pone.0050093

**Published:** 2012-12-18

**Authors:** Xin Li, Rebecca J. Stones, Haidong Wang, Hualiang Deng, Xiaoguang Liu, Gang Wang

**Affiliations:** 1 Nankai-Baidu Joint Laboratory, College of Information Technical Science, Nankai University, Tianjin, China; 2 Clayton School of Information Technology, Monash University, Victoria, Australia; 3 School of Mathematical Sciences, Monash University, Victoria, Australia; 4 College of Software, Nankai University, Tianjin, China; Northwestern University, United States of America

## Abstract

A motif in a network is a connected graph that occurs significantly more frequently as an induced subgraph than would be expected in a similar randomized network. By virtue of being atypical, it is thought that motifs might play a more important role than arbitrary subgraphs. Recently, a flurry of advances in the study of network motifs has created demand for faster computational means for identifying motifs in increasingly larger networks. Motif detection is typically performed by enumerating subgraphs in an input network and in an ensemble of comparison networks; this poses a significant computational problem. Classifying the subgraphs encountered, for instance, is typically performed using a graph canonical labeling package, such as Nauty, and will typically be called billions of times. In this article, we describe an implementation of a network motif detection package, which we call *NetMODE*. NetMODE can only perform motif detection for 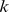-node subgraphs when 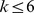, but does so without the use of Nauty. To avoid using Nauty, NetMODE has an initial pretreatment phase, where 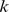-node graph data is stored in memory (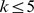). For 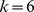 we take a novel approach, which relates to the Reconstruction Conjecture for directed graphs. We find that NetMODE can perform up to around 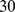 times faster than its predecessors when 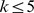 and up to around 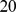 times faster when 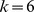 (the exact improvement varies considerably). NetMODE also (a) includes a method for generating comparison graphs uniformly at random, (b) can interface with external packages (e.g. R), and (c) can utilize multi-core architectures. NetMODE is available from netmode.sf.net.

## Introduction

A *network motif* in a network 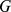 is a connected graph 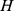 that occurs significantly more frequently as an induced subgraph than would be expected in a “similar” random network. The term “network motif” was coined by [1], [2], who discovered that they occur in several biological and artificial networks. Recently, network motifs have been found in a vast range of networks, and, in some cases, have been identified as functionally important. One prominent example is the 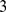-node feed-forward loop 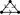 in the E. coli transcription factor network [3]. For further reading, see [4], [5]. See also Supporting Information S1 for an introduction to the graph theory concepts used in this paper.

### Definition in practice

In practice, the detection of network motifs is typically implemented in the following way.

- *Step 1*: Compute the number of times that 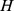 occurs in 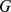 as an induced subgraph, call this number 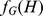. We will assume that distinct copies of 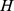 may overlap (although, this is topical).
- *Step 2*: Generate an ensemble 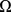 of random graphs “similar” to the input network.
- *Step 3*: For each graph 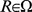, we compute the number of times 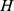 occurs in 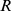, which we will denote 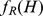.
- *Step 4*: Compute the probability 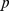 (called the 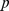-*value*) that a similar graph 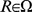 contains the same or more copies of 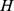 than 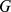. If the estimate for 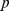 is less than some user-defined threshold 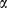, we declare 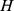 a network motif.

Performing these steps for all 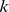-node connected subgraphs will be referred to as performing a 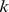-node *subgraph census*.

In some motif detection programs, a *Z-score* is used instead of, or together with, the 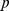-value above; it is given by 
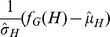
 where 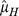 and 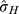 are the sample mean and standard deviation of the dataset 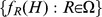. However, we caution the reader that, in this context, the use of a “Z-score” does not imply a corresponding normal distribution.

Typically, a graph is described as *similar* to 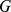 if it has the same vertex set as 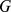 and if its vertices have the same in-degrees and out-degrees as in 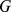. However, the treatment of loops and bidirectional edges can be addressed in different ways. We will describe how we address these issues in the “Modifications” section. In any case, we will refer to the similar graphs in 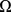 as the *comparison* graphs.

In practice, additional criteria are added, such as a minimum value for 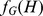. Instead of frequency, concentration might also be considered [6], that is, the proportion of 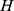 amongst all 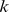-node connected induced subgraphs in the network. The decision of which statistic to use can have a significant impact on whether or not 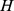 is declared a motif.

### Software

Many packages have been developed that enable motif detection. Mainstream packages, those that can perform a 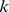-node subgraph census, include Mfinder [7], MAVisto [8], [9], FanMod [6], [10], [11], MODA [12], and Kavosh [13]. This is the family of network motif detection packages to which our program, NetMODE, belongs.

Of these, we will focus on FanMod and Kavosh, which are the most relevant: Mfinder and MAVisto are largely subsumed by FanMod; the authors of MODA have asked us to consider Kavosh instead (via private communication). We find Kavosh is typically faster than FanMod (see Table 1).

**Table 1:** Speedup of NetMODE vs. Kavosh.

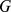	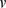	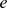	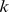	run-time (sec.)	K-speedup	
				Kavosh	serial	4-core
1	67	182	3	0.5	0.5	1.8
Social			4	1.5	1.5	2.1
			5	8.5	3.2	2.4
			6	50.5	1.5	4.9
2	672	1276	3	3.9	1.0	7.1
E. coli			4	11.7	3.5	20.0
			5	79.6	6.2	15.7
			6	752.1	4.2	16.1
3	688	1079	3	10.5	4.4	13.2
Yeast			4	214.2	17.7	74.6
			5	5990.8	31.8	123.5
			6	119359.3	11.4	46.1
4	50	2540	3	20.7	7.6	27.0
Complete graph			4	460.7	25.5	107.6
			5	6971.7	31.7	122.1
			6	88025.3	20.4	81.0

G-Tries [14]–[17] (see also [18]) is a data structure whose authors claim impressive speedups vs. FanMod. We do not include G-tries in the experiments in this paper since the G-tries data structure comprises only part of motif detection, although we give a theoretical analysis in the Theory section. Moreover, only recently has a “preliminary” program, called gtrieScanner, that implements the G-tries data structure become available; we make some remarks about gtrieScanner in the Conclusion.

There is also a range of other algorithms and packages that have some specialized functionality, or functionality related to motif detection, such as NeMo [19], which considers non-induced subgraphs.

There are also several papers that compare algorithms and software for network motif detection [20]–[26].

## NetMODE

We will now introduce our network motif detection package NetMODE, **Net**work **MO**tif **DE**tection, designed to improve upon Kavosh for 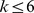.

### Modifications

NetMODE began development as a modified version of Kavosh, but over time was modified so substantially that we no longer consider it a Kavosh variant. We will now explain the differences.

#### Canonical labeling

In packages such as FanMod and Kavosh, the highly-optimized graph isomorphism package Nauty (available from cs.anu.edu.au/bdm/nauty/) is used to provide canonical labels for all of the 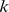-node subgraphs encountered. Typically, they make millions, or even billions, of calls to Nauty. However, in small cases, the number of isomorphism classes of directed graphs is not overly large. Consequently, Nauty is called to perform the exact same task many times over. Moreover, often the majority of 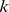-node subgraphs encountered fall into a handful of isomorphism classes.

NetMODE instead stores all canonical labels in memory. This scheme works easily for 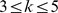, but for 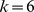 we need to be a bit more clever, and for 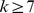, this scheme seems totally impractical. Any significant run-time improvement achieved by NetMODE is the result of this preprocessing scheme.

#### Generating similar graphs

NetMODE has several methods for sampling similar graphs; which one to use should be determined based on the input network.

We employ several variants of a switching method similar to that described in [27]. The switching operation is quite simple: two edges 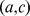 and 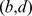 are randomly chosen, and are replaced by 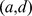 and 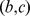 provided no loops or parallel edges are introduced.

Kavosh uses a modified version of this switching process which has some unfortunate drawbacks. FanMod has three different options for the handling of bidirectional edges. In NetMODE, we modify Kavosh's switching method to allow FanMod-like functionality while still using Kavosh's edge selection method. Additional details regarding comparison graph generation methods used in Kavosh, FanMod, and NetMODE are given in Supporting Information S1.

In all of the above cases, the random graphs are sampled from a non-uniform distribution [28]. Furthermore, Ginoza and Mugler [29] discussed how the switching operations can change subgraph counts in a highly correlated manner. However, in practice, the switching method seems reasonable enough (when compared to uniform sampling). Note that different switching methods will incur different run-times.

An alternative method for sampling similar graphs has also been enabled in NetMODE. This option generates similar graphs uniformly at random, but is typically much slower than the switching methods. It implements the “local constant mode” using a variant of the Configuration Model. In some cases, it is extremely slow, but if the user finds it infeasible for a given input network, they may revert to a switching method. A related “stubs” method was implemented in Mfinder.

#### Subgraph enumeration

Kavosh utilizes a “revolving door” routine to iterate through combinations of vertices. (The “revolving door” routine minimizes the number of changes between each iteration, with the aim to improve computational efficiency.) NetMODE utilizes Kavosh's subgraph iteration procedure, except it does not use the revolving door algorithm; it does not seem to have any significant benefit for our purposes.

### Structure

When NetMODE is run, it will first enter a *pretreatment phase*. If 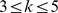, then a list of all 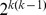 possible loop-free 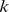-node directed graphs are stored in memory along with their canonical labels (obtained via a brute-force search). For 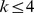, we experience negligible overhead, while for 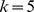, we experience around 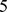 seconds overhead (this will differ on varying platforms). For 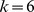 this approach is infeasible, so we take an alternative approach that involves a special case of a variant of the Reconstruction Conjecture in graph theory. An introduction to the Reconstruction Conjecture is given in Supporting Information S1.

From a 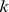-node graph 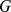, we can generate 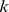 induced subgraphs on 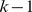 vertices, called *cards*, by deleting one vertex 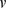 (and the edges that have 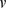 as an endpoint). The multiset of cards from a given graph is called the *deck*, which we will denote 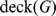. Note that if 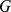 and 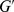 are isomorphic graphs, then 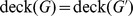. By two independent computer searches we find that the converse is true for 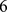-node directed graphs barring a few exceptions, as detailed in Theorem 1.

**Theorem 1** *If* 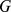 and 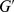 *are two non-isomorphic loop-free* 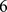
*-node directed graphs, then* 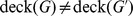, *except when* 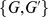 *belongs to the following list of exceptions:*
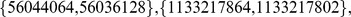

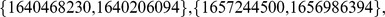

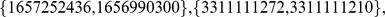

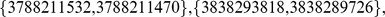

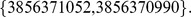
Theorem 1 asserts that, with a few exceptions, loop-free 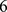-node directed graphs are determined (up to isomorphism) by their 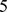-node induced subgraphs. Thus, we instead store in memory the canonical labels for 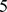-node directed graphs, and use this data for canonical labeling 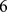-node subgraphs. In Theorem 1, graphs 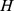 are listed as their *graphID*, the number defined when the adjacency matrix is read as a binary number; it is also *canonical*, the minimum such number in the isomorphism class. The computation required to prove Theorem 1 has been performed independently by the authors Li and Stones (the code used has also been made available).

When running NetMODE for 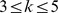, on both the input network and the random comparison graphs, we iterate through all 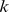-node subgraphs 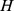, and simply increase the count corresponding to its canonical label. Running NetMODE for 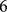-node subgraphs is more complicated and involves two distinct stages. In Stage 1, we process the input network. We start with an empty list 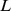, and as the algorithm proceeds:

- If 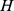 is an exception, we account for it separately (details are given in the Supporting Information S1), otherwise we continue.
- If 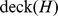 does not appear in 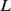, we add a new entry to 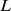 (with count 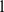). Otherwise, 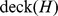 appears in 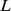, so we simply increase its count. Searching through 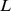 is facilitated by a hash function; its details are described in the Supporting Information S1.

In Stage 2 we process the comparison graphs. When a 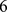-node subgraph 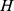 is found, we compute 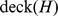 and search for its hash value in 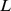. If found, then we search for 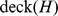 amongst all entries with that hash value, and if 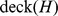 is found, we then increase its count. Otherwise, if its hash value or 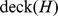 is not found in 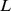, we do nothing.

For 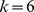, note that we store, and hence return, the first graph isomorphic to 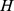 found in the input network, which is not necessarily in canonical form. Note also that, in all cases, NetMODE only returns the counts of subgraphs that are found in the input network.

### Other features

#### Usability

The theory of network motifs is relatively new and has received some criticism, e.g. [30]. Consequently, we should be careful when making claims relating to network motifs. With this in mind, we have included a *verbose mode* in NetMODE, where we return the number of subgraphs isomorphic to 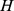 in both the input network 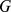 and the comparison graphs. This allows the user access to all of the data encountered during run-time, so that the user can perform their own independent analysis. Typically, this is a large amount of data, so verbose mode is intended for analysis via a separate program.

NetMODE uses stdin/stdout so that it can interface with well-established packages, such as R. We also provide some R code that can be run to enable interfacing with NetMODE in verbose mode (using the igraph extension). The user can therefore use R to e.g. draw motifs, check whether or not sufficiently many comparison graphs have been used, check whether the comparison graph counts are approximately normally distributed, and so on.

We also introduce a *burnin* feature, whereby the first few comparison graphs generated by the switching operation are discarded, resulting in a better ensemble of comparison graphs. The benefit of using burnin is that it essentially allows the switching process to start at a random starting point (rather than the input network); its use is standard practice in MCMC algorithms. In general, it is unclear what amount of burnin to use, but we found that 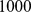 discarded comparison networks seems reasonable in most cases.

#### Parallelism

We also design NetMODE to be capable of utilizing parallel processing in multi-core computers. We use a basic coarse-grained parallelism where an individual thread is responsible for performing subgraph census on one of the comparison graphs. Parallelism is only invoked for the comparison graphs, whereas the input graph is processed in serial. At this level, it is an embarrassingly parallel problem, in that no communication is required between individual threads once they are created. Message passing is handled by the pthread library. Each comparison graph computation is added to a queue, which is managed by a *thread manager*. The thread manager limits the number of simultaneous threads to some user-defined threshold.

## Experimental Results

In this section we report the experimental results obtained from testing the various algorithms described in this paper.

### Experimental setup

Unless otherwise specified, the platform used in these experiments is: AMD Phenom II X4 945 (4 cores) with 2
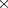
2 GB RAM running Red Hat Enterprise Linux 5.

The number of similar graphs generated for comparison is always 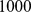 (we do not endorse this number of comparison graphs be used in practice; users should decide for themselves based on the input network and value of 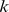), and we use zero burnin. Each experiment is performed only once, since differences in run-times are negligible.

In its downloadable form, Kavosh wastes a lot of time printing to the screen and writing to files. To obtain a more reasonable comparison, all computations involving Kavosh are performed using a modified version in which printing is disabled; similarly we disable writing to the disk. The difference can be significant: e.g. 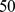 minutes vs. 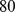 seconds on the E. coli dataset, 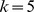.

### Speedup

We define the *K-speedup* (resp. *F-speedup*) of NetMODE to be the number of times faster NetMODE is than Kavosh (resp. FanMod) when processing a given dataset. All variables (platform, number of comparison networks, etc.) are kept the same, except possibly for the number of threads (which we will explicitly highlight, when relevant).

In Table 1, we list the K-speedup in a range of instances. For Table 1 we use the “fixed bidirectional edges” switching method, since it matches Kavosh's switching method. For the given input network, we use 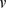 to denote the number of nodes and 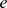 to denote the number of directed edges. We test NetMODE using four input networks: (a) a social network, (b) the metabolic pathway of E. coli, (c) the transcription network of S. cerevisiae (yeast), and (d) the complete directed graph on 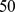 vertices. Input networks (a), (b), and (c) were used in testing Kavosh and were packaged with the Kavosh source code. No description other than “a real social network” was given for input network (a) in [13].

The fourth input network was chosen since it has some easy-to-compute properties, e.g. in each of the 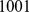 networks (the input network and all 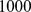 comparison networks), there are exactly 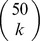 copies of 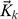. Therefore, the number of calls to Nauty by Kavosh is 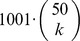.

Table 2 gives some example run-times for FanMod and NetMODE under various switching methods. Notation: F = fixed bidirectional edges; NR = no regard; GC = global constant; LC = local constant; ULC = uniform local constant. In some instances, ULC mode is impractically slow.

**Table 2:** Run-times (sec.) for Kavosh, FanMod, and NetMODE under various switching methods.

	Yeast; 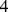 -node subgraph census				
	F	NR	GC	LC	ULC
Kavosh	214.2	–	–	–	–
FanMod	–	318.0	319.0	318.0	–
NetMODE	12.1	12.6	12.0	12.3	–
NetMODE 4-core	2.9	2.9	3.0	3.0	–

### Scalability

In this section we describe the results of experiments designed to test the scalability of NetMODE. For these experiments, we use the local constant switching method (LC), which is probably the most reliable of the possibilities. Other switching methods can take longer, cf. Table 2.

#### Size of input

**Figure 1:**
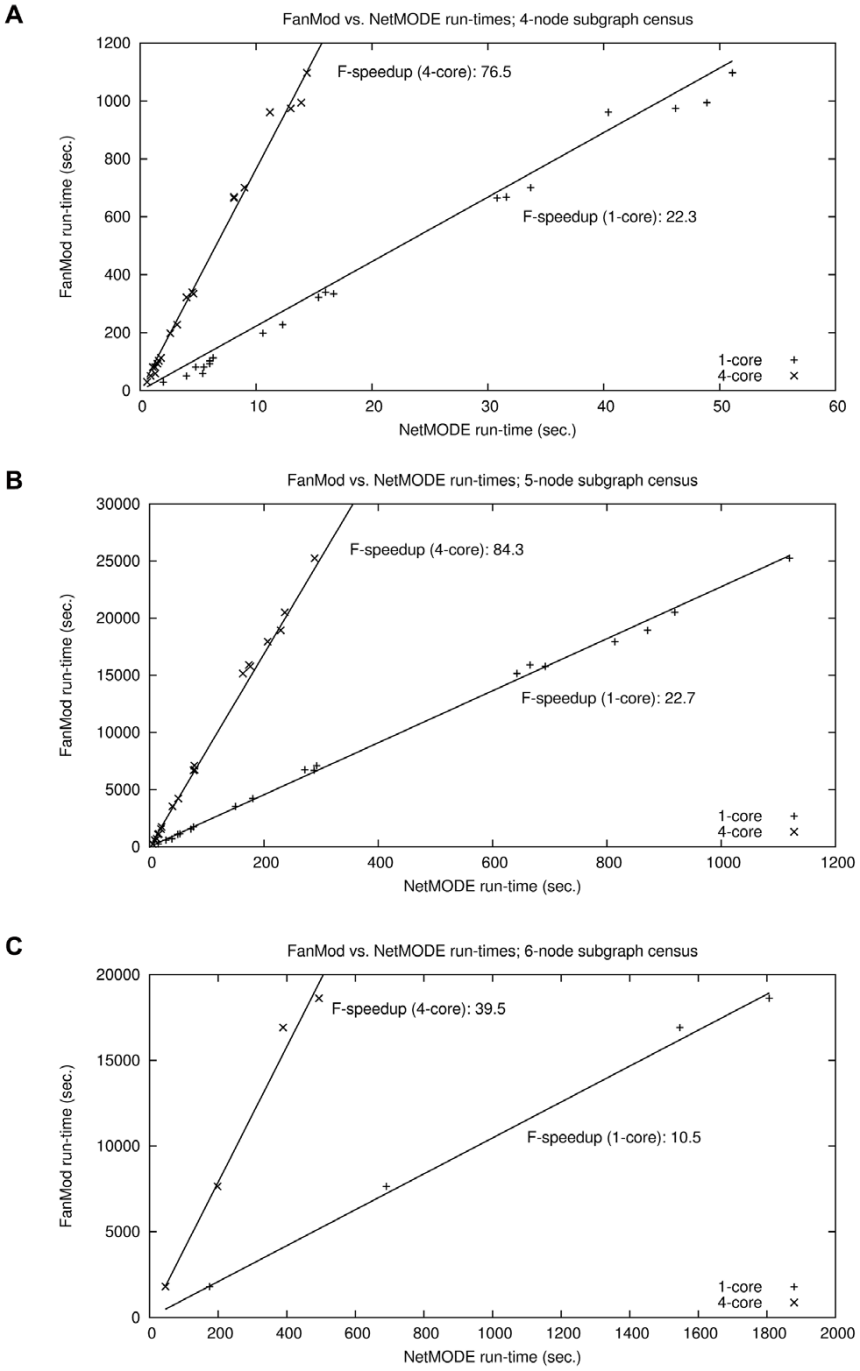
Run-times of NetMODE and FanMod on various protein structure networks.

Figure 1 plots the run-times of NetMODE and FanMod in a range of circumstances. In these experiments, we use “protein structure networks” (as defined in [1]), for a range of proteins, whose structures were obtained from the Protein Data Bank [31]. The protein ID numbers are: 1IEG, 1DNP, 1VR0, 1F3U, 1OLM, 1HI9, 2IDB, 2VV5, 7AHL, 1B65, 2UUB, 1V54, 1HBN, 1SUV, 1K1E, 1UW6, 1RVV, 1KP8, 1GR5, 2FUG, 1TZN (although, only 1IEG, 1DNP, 1VR0, and 1F3U were used for the 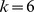 experiments). These networks are undirected, where undirected edges are treated as bidirectional edges. In several instances, we were unable to run Kavosh for these networks, so we use FanMod for the run-time comparison. The networks are also available for download with NetMODE.

### Number of cores

**Figure 2:**
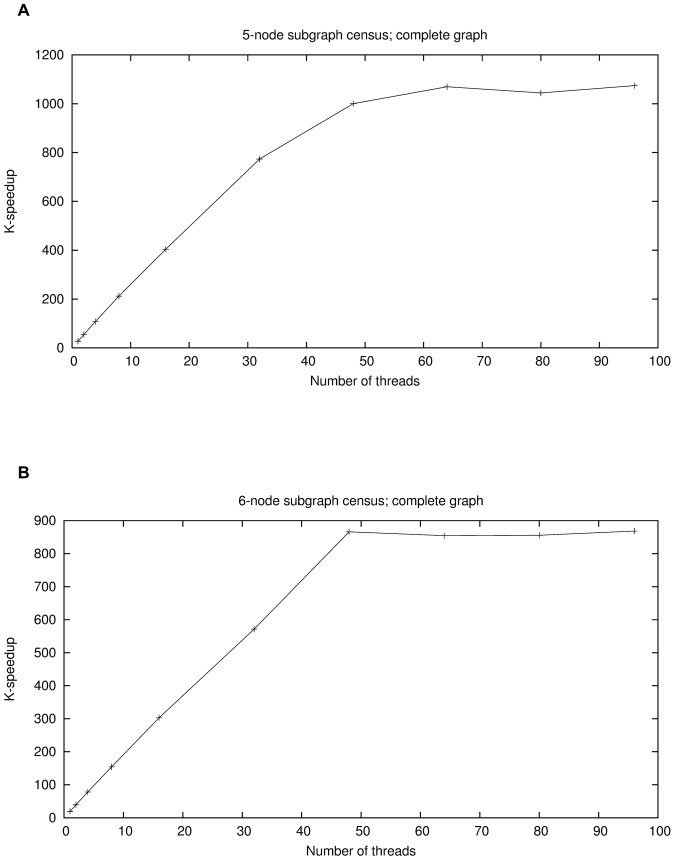
Speedup of NetMODE vs. Kavosh as the number of threads varies. Kavosh took 713 seconds and 134670 seconds, respectively.

Figure 2 plots the run-time by the multi-core version of NetMODE as the number of utilized CPU cores varies. To perform this experiment, we switch to a platform with more cores: Red Hat Enterprise Linux 5; AMD Opteron Processor 6168 (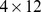 cores); 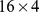
G RAM. The input network was the complete directed graph on 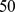 vertices.

### Accuracy

Since NetMODE allows uniform local constant (ULC) random comparison graph sampling, we can inspect the accuracy of NetMODE's results. In Table 3 we list some concentration-based Z-scores returned by Mfinder in local constant mode, estimated using 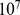 samples (denoted LCs), FanMod in local constant (LC) mode, and NetMODE in both LC and ULC modes. We also list some frequency-based Z-scores returned by Mfinder in LC mode, and NetMODE in both LC and ULC modes.

**Table 3:** Concentration and frequency Z-scores returned by FanMod, Mfinder, and NetMODE; E. coli network, 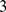-node subgraph census.

	Concentration				Frequency		
	Mfin.	FanM.	NetM.	NetM.	Mfin.	NetM.	NetM.
gID	LCs	LC	LC	ULC	LC	LC	ULC
6	−3.65	−3.35	−3.49	−3.66	−11.94	−11.60	−11.86
12	−4.10	−4.25	−4.54	−4.27	−13.13	−12.60	−12.02
14	2.66	2.98	2.50	2.55	−10.18	−9.49	−9.65
36	−5.96	−6.09	−6.14	−6.34	−12.80	−12.70	−13.17
38	16.34	16.40	16.75	16.75	15.47	15.59	16.08
46	1.12	1.22	1.17	1.25	1.20	1.09	1.18
74	3.55	3.76	3.26	3.42	−8.92	−8.33	−8.61
78	−16.56	**−46.11**	−15.97	−16.24	−21.94	−21.60	−20.62
98	5.16	5.01	5.08	4.73	5.42	4.95	4.53
102	3.42	3.06	3.32	3.25	3.21	2.80	3.09
110	11.91	11.84	11.65	11.62	11.71	11.56	11.14
238	19.05	**98.99**	18.37	18.98	18.83	18.77	18.23

## Discussion

### Experiment

In the experiments in this paper, the primary focus is on run-time, since this is the main advantage for the end user. Memory requirements will typically be easily met, even on modest computers.

From our experiments, we find that Kavosh is usually faster than FanMod (i.e. the speedup vs. FanMod will typically be slightly greater than a speedup vs. Kavosh). To put this into a broader context, FanMod admits a host of functionality, such as allowing colored graphs as input networks, and the use of a sampling method (rather than complete enumeration). Neither Kavosh nor NetMODE have these features. NetMODE specializes on one specific task, and is faster than FanMod at this one task. However, NetMODE also offers multi-core parallelism, a uniform method for generating comparison graphs, and the ability to access the comparison graphs' subgraph counts, which are all important features that are absent from FanMod.

Tables 1 and 2 give run-times and speedups of NetMODE in a range of circumstances. We see that NetMODE can attain substantial speedups vs. Kavosh and FanMod. We also see a noticeable drop in speedup from 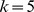 to 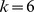 as NetMODE switches to a different mode. Table 2 also compares the run-times of NetMODE and FanMod under different methods for generating comparison graphs. We see that, while some switching methods are slower in NetMODE, they are also correspondingly slower in FanMod.

Figure 1 shows that the speedup achieved by NetMODE is roughly constant with the size of the input network. Figure 2 shows a near-linear increase of speedup with the number of cores utilized. In some instances, we see speedups of over 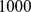, and expect that this would be increased further if we were to use more cores.

We give a comparison of Z-scores returned by FanMod, Mfinder, and NetMODE in Table 2. We see that NetMODE's results resemble that of the uniform null model. FanMod's results, which typically resemble the uniform null model, are sometimes wildly different. Specifically, the graphs with graphID 78 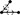 and 238 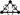 have surprising Z-scores. We can exclude the possibility of this discrepency being the result of a bug with NetMODE since NetMODE and Mfinder's results are comparable. This indicates a bias in FanMod's results in some cases. Further experimentation relating to FanMod's bias is given in Supporting Information S1.

### Theory

#### Maximum theoretical speedup

If 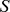, 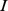, and 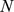 are the run-times of the components “generate similar graphs”, “iterate through subgraphs”, and “canonically label subgraphs”, then NetMODE could achieve a speedup vs. Kavosh (or FanMod) of at most
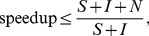
(1)since these programs share the 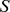 and 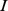 components.

Methods for decreasing 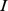 (along with 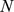) are already available, such as estimating the concentration of subgraphs via sampling, or searching for only a single 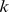-node subgraph. These approaches offer a trade-off of accuracy or scope for run-time. These methods will still have a theoretical best speedup of 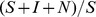.

An alternative approach is to compute the 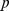-values analytically, which is not easy to do with the established definition of a “similar” graph. Such an approach was use in NeMo [19], but for a different distribution of comparison graphs.

Some other programs avoid the use of Nauty by using symmetry breaking. These include the program by Grochow and Kellis, which searches for one 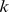-node subgraph at a time, and both MODA and G-tries, which utilize a tree-like data structure to guide their subgraph searching (although in dissimilar ways). Another approach was offered by Baskerville and Paczuski [32], which uses a heuristic based on vertex invariants; it is able to distinguish all isomorphism classes of undirected graphs for 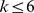 and most isomorphism classes for 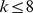.

Another bound dominates when 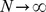 and 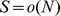. In these instances, if 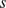 and 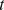 are respectively the average times taken to canonically label a given subgraph by NetMODE and Kavosh, then the speedup will be bounded above by 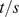. To estimate 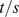, we ran NetMODE and Kavosh on the input network 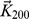 (with 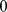 comparison graphs), which gave speedups of
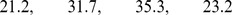
for 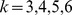, respectively.

#### Remarks on G-tries

Ribeiro and Silva [15] claimed speedups of G-tries vs. their own version of FanMod of over 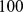 in some cases. However, there are several properties of their experimental design and results that indicate these results would not be applicable to an end user.

Since G-tries describes a data structure rather than a full program, we will discuss Program G, a hypothetical program which incorporates G-tries, as described by [15].

#### G-tries vs. NetMODE

In order to perform a 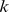-node subgraph census, Program G generates random comparison graphs and iterates through all 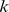-node connected subgraphs, thus its run-time contains the components measured by 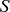 and 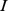. Any significant speedup of Program G vs. FanMod is therefore achieved wholly by reducing 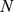 (i.e. by avoiding the use of Nauty), but this is achieved in a much more straightforward fashion in NetMODE.

#### Excluding 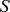


The experiments for benchmarking G-tries were designed to test only the components improved by G-tries. As such, the authors have excluded the time it takes to generate similar graphs (i.e. 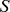).

To illustrate the discrepancy, in the power network tested in [15], we find that 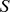 amounts to more than 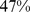 and 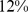 of the total run-time in FanMod, when performing a 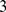-node and 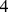-node subgraph census, respectively (undirected graphs, complete enumeration). Consequently, it is theoretically impossible for Program G to achieve a speedup vs. FanMod of more than around 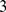 or 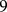, respectively, in this example (which is much less than the 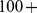 speedups reported). This issue reduces as 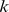 increases, since 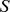 becomes relatively smaller.

#### Measurements

The run-times by the version of FanMod used in [15] appear to be much larger than can be achieved by an authentic version of FanMod. For example, in Table 4 we list the average run-time (sec.) per comparison graph to perform a 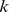-node subgraph census given by [15] on the “power” network, along with that achieved by an authentic version of FanMod run on a slower computer (Windows XP, 1.66 GHz, 1 GB RAM).

**Table 4:** Examples of run-time discrepency between G-tries's FanMod and the authentic version of FanMod.

k	G-tries's FanMod	authentic FanMod	articial speedup
3	0.91	0.052	≥17.6
4	3.01	0.202	≥14.9
5	12.38	1.043	≥11.9

### Parallelism

NetMODE can utilize multi-core architecture, such as in many modern computers. Considering that multi-core hardware has been widespread for some time (particularly in bioinformatics), the inability of other programs to utilize parallel hardware is a serious limitation.

Figure 2 illustrates that the multi-core version achieves a near-linear speedup (which should be expected in such embarrassingly parallel problems). The computer used in this experiment has 48 CPU cores, so increasing the number of threads used beyond 48 did not achieve anything significant.

We also made an attempt to accelerate the subgraph searching method on a graphics processing unit (GPU). We use NVIDIA's CUDA parallel computing architecture, and testing was performed on the NVIDIA GeForce GTX 480. We tried two approaches:

1. We collect a number of graphs and distribute them to the GPU's blocks; each kernel performs a separate subgraph searching procedure. After the kernel exits we combine the results from several graphs.
2. We search a single graph in one call, requiring each block to search a subset of the vertices. When one random graph is generated, we launch a kernel. After the kernel exits we combine the results from that single graph, and move onto the next graph. This method was also carefully optimized with the kernel stream.

Unfortunately, neither method displayed significant performance. This comes as a surprise, considering how useful the GPU is for detecting “sequence motifs” (see e.g. [33]–[35]). We believe this is mainly a result of the divergence within the subgraph searching procedure. The GPU is optimized for performing similar operations in different threads, so the differences between the searching procedure (caused by the randomness of the comparison graphs) together with the enormous memory requirements slow down the kernel. To help future researchers, these programs are made available for download with NetMODE.

### Combinatorial explosion

There are extremely many connected digraphs on 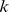 nodes for large 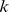. The number of non-isomorphic connected 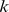-node digraphs is given by Sloane's A003085 and the number of non-isomorphic connected 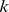-node undirected graphs is given by Sloane's A001349; see oeis.org. The first few values are listed in Table 5.

**Table 5:** The number of 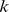-node connected digraphs and undirected graphs, respectively.

k	A003085	A001349
1 1 1	1	1
2	2	1
3	13	2
4	199	6
5	9364	21
6	1530843	112
7	880471142	853
8	1792473955306	11117

This property is sometimes referred to as “combinatorial explosion”. If we were to run e.g. a 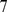-node subgraph census on a directed graph, the sheer number of non-isomorphic 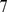-node digraphs will give rise to many estimated 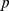-values of 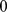 (the reader can readily verify this with FanMod). Unless we use extremely many comparison graphs, mixed in the list of subgraphs with an estimated 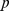-value of 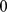 will be many insignificant subgraphs. In fact, this problem is still a significant concern at 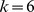.

An alternative is to use Z-scores instead of 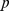-values, but this was shown to be an unreliable statistic by Picard et al. [36] (and more about this will appear in [37]); we include Z-scores in NetMODE mainly for reasons of comparison. Thus, to distinguish between the subgraphs with an estimated 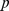-value of 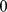, the user should repeat the experiment using a larger number of comparison graphs. Practically, this is a double blow, not only does the computational time increase rapidly with 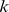, but also increases linearly with 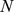 (which should be chosen to increase rapidly with 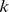).

In NetMODE, we enable the user to use more comparison graphs through parallelism (and by simply being faster than previous programs). FanMod (and Mfinder) instead offers the option of estimating subgraph concentrations via subgraph sampling (rather than complete enumeration).

## Conclusion

We have implemented a network motif detection package, NetMODE, designed to improve on Kavosh and FanMod by minimizing the time taken for canonical labeling. While other packages, such as FanMod, offer a host of functionality, NetMODE's function is more specialized: it consideres only uncolored graphs, performs only complete enumeration and searches for 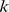-node motifs for 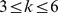. The goal for NetMODE is to perform this task faster than its predecessors, which has been achieved through a preprocessing scheme.

We have also designed NetMODE to be functional on multi-core parallel architectures. Running NetMODE on multi-core hardware should be straightforward to use even for less computer savvy users: just add e.g. “-t 2” to the command line to use two threads. For portability, we have developed NetMODE to use stdin/stdout, and allow interfacing with the popular R statistical package. Since NetMODE is substantially faster than FanMod (and can be run in parallel), it offers the ability to search for 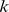-node motifs for 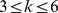 (using complete enumeration) within larger input networks than FanMod would practically cope with.

One apparent technique for practically extending NetMODE's functionality to 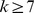 is, in the preprocessing phase, store in memory only the canonical labels of the subgraphs that are likely to be encountered (reverting to calling Nauty whenever other subgraphs are encountered). In this case, which subgraphs to preprocess could be determined from testing, or from an auxiliary file. However, we have not explored this avenue in NetMODE since we consider the problem of combinatorial explosion to be overwhelming at this stage, and, in any case, the majority of research interest so far has been for subgraphs with 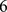 or fewer nodes.

Brendan McKay suggested another way to improve preprocessing times: generating and storing in memory only the 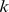-node digraphs whose degree sequences are in “order” with respect to the vertex labels (for directed graphs, the “order” could be the lexicographic order on in-degree/out-degree pairs). In this scheme, when we encounter a 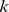-node subgraph, we first permute its vertices so that its degree sequence is in order, then recall its canonical label from memory.

### G-tries run-time

After the submission of this paper, gtrieScanner, a network motif detection package based on G-tries was released on Ribeiro's webpage (http://www.dcc.fc.up.pt/gtries/). This gave the present authors an opportunity to compare the two programs. Recall that both programs improve upon previous methods by minimizing the canonical labeling component, i.e. 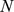 in (1).

Ribeiro commented that the current release of gtrieScanner (version 0.1) is a “preliminary” version of a program that implements the G-tries data structure. Thus, we feel it would be misleading to give a full-blown comparison of this version of gtrieScanner vs. NetMODE. However, we make the following observations:

- For the experiments we performed, the relevant subgraph counts, Z-scores, and 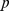-values between NetMODE and gtrieScanner were consistent.
- For performing a 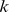-node subgraph census with 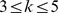, the run-times of gtrieScanner and NetMODE were comparable. This suggests that both methods have improved upon previous methods by minimizing the time taken to canonically label subgraphs. Differences in e.g. preprocessing times and memory usage causes some fluctuation between the run-times of gtrieScanner and NetMODE.
- For a 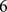-node subgraph census, gtrieScanner performed much faster than NetMODE for undirected networks (but returned a segmentation fault for directed networks). Moreover, unlike NetMODE, gtrieScanner can also perform 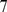-node to 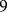-node subgraph census for undirected networks.
- For large input networks, gtrieScanner used a considerable amount more memory than NetMODE. We gleam from the source code, that gtrieScanner stores e.g. the input network's adjacency matrix in memory, thereby requiring 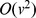 memory, where 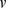 is the number of nodes in the input network. This approach is not scalable, and gtrieScanner is virtually unusable for graphs with 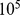 or more nodes.

Note also that, unlike NetMODE, gtrieScanner is not (yet) a parallelized program.

## Supporting Information

Supporting Information S1 A document giving an introduction to the concepts required to understand this paper along with an outline of how to use NetMODE.
(PDF)
