# Correction: NetMODE: Network Motif Detection without Nauty

**DOI:** 10.1371/journal.pone.0231195

**Published:** 2020-03-26

**Authors:** Xin Li, Rebecca J. Stones, Haidong Wang, Hualiang Deng, Xiaoguang Liu, Gang Wang

## Notice of republication

This article was republished on March 16, 2020, to correct the author list. Please download this article again to view the correct version.

## References

[1] Li X, Stones RJ, Wang H, Deng H, Liu X, Wang G (2012) NetMODE: Network Motif Detection without Nauty. PLoS ONE 7(12): e50093 10.1371/journal.pone.0050093 23272055 PMC3525646

