# Supplementary material for: NetMODE: Network Motif Detection without Nauty
Source: PLoS One. 2012 Dec 18;7(12):e50093. doi: 10.1371/journal.pone.0050093 (PMC3525646; doi:10.1371/journal.pone.0050093)
Supplement: Supporting Information S1 — A document giving an introduction to the concepts required to understand this paper along with an outline of how to use NetMODE. (PDF) [file pone.0050093.s001.pdf]

NetMODE: Network motif detection without Nauty; supplementary material

Xin Li, Douglas S. Stones, Haidong Wang,
Hualiang Deng, Xiaoguang Liu, Gang Wang

June 28, 2012

This document is supplementary material for the main paper: "NetMODE: Network motif detection without Nauty." In particular, we introduce the Reconstruction Conjecture and discuss the generation of random similar graphs. We also describe a basic method for calling NetMODE from R, which allows the user to analyze the data in their own way. We also include the results of some experiments not described in the main text.

Contents

1 Graph theory **2**
- 1.1 Introduction 2
- 1.2 The Reconstruction Conjecture 4
  - 1.2.1 Application in NetMODE 5
- 1.3 Similar graphs 5
  - 1.3.1 Kavosh and FanMod 6
- 1.4 Configuration Model 7
  - 1.4.1 Uniformity 9
  - 1.4.2 Comparison graphs in NetMODE 10

2 Using NetMODE **11**
- 2.1 R 11
- 2.2 Command line 15

3 Further testing of NetMODE **16**
- 3.1 Number of comparison graphs 16
- 3.2 E. Coli transcription factor network 16
- 3.3 Output comparison with Kavosh and FanMod 18
  - 3.3.1 Bias in FanMod 18

1 Graph theory

In this section, we give an introduction to the graph theory concepts required to understand the concepts in the main text, along with an introduction to the Reconstruction Conjecture and the variant of the Configuration Model used for generating similar graphs uniformly at random.

1.1 Introduction

A *graph* is a collection of *nodes* (or *vertices*) together with a collection of *edges* joining pairs of nodes. Typically graphs are denoted $G = (V, E)$ where $V = V(G)$ is the set of nodes and $E = E(G)$ is the set of edges. The edges (or *arcs*) in a *directed* graph (or *digraph*) have an order, that is, an edge $(i, j) \in E(G)$ is different to $(j, i) \in E(G)$. Thus it is possible for both (i, j) and (j, i) to occur in the same directed graph, which we refer to as a *bidirectional* edge. We tend to think of a directed edge (i, j) as coming out of node i and going into node j. In an *undirected* graph, the edges do not have an order, so an edge $\{i, j\} = \{j, i\}$. In either case, i and j are called the *endpoints*.

An undirected graph can be considered to be a directed graph in which undirected edges $\{i, j\}$ are replaced by directed edges (i, j) and (j, i). This concept is illustrated below. If both edges (i, j) and (j, i) occur in the graph, then we typically think of them combined into a single bidirectional edge. NetMODE focuses on directed networks since they are therefore more general.

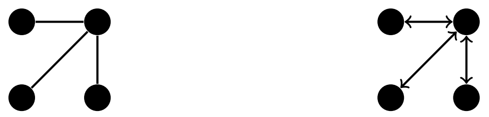

an undirected graph; the equivalent directed graph

Loops (or *self-loops*) are where an edge (i, i) from a vertex to itself exists. We caution the reader that, for practical reasons, NetMODE ignores *loops*. A more general class of graphs include *parallel edges*, where edges (i, j) occur more than once. NetMODE also ignores parallel edges.

In standard usage, a *subgraph* of $G = (V, E)$ is a graph $H = (V', E')$ for which $V' \subseteq V$ and $E' \subseteq E$ and the edges in E' have endpoints in V'. A subgraph is called *induced* if $E' = \{(i, j) \in E : i \in V' \text{ and } j \in V'\}$, that is, it includes every edge in the original graph whose endpoints are both in V'. A graph is called *connected* if there is a path between any two vertices ignoring edge directions (in graph theory, this is referred to as *weakly connected*).

Most network motif detection software count only connected induced subgraphs (and many papers omit mention of the requirement that they be connected and induced). MODA [18], RAGE [12, 13], and NeMo [10] are exceptions here, as they can perform non-induced subgraph enumeration (see also [1]).

Below are three graphs that highlight the difference. The graph in the center is a subgraph of the left graph, however it is neither connected nor induced. The graph on the

right is a (weakly) connected and induced subgraph.

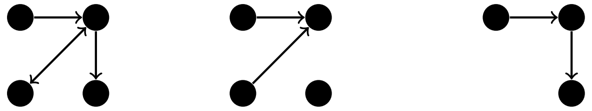

a directed graph; a subgraph; an induced subgraph;

A graph is called *labeled* if its vertices have been assigned distinct *labels*; often these labels are $\{0, 1, \ldots, n-1\}$ for an n-node graph. A permutation α can be used to "act on" a labeled graph G, specifically, αG is the graph formed by relabeling each vertex i in G by $\alpha(i)$. We call α an *isomorphism* and say G and αG are *isomorphic*. In fact, if G has $|V| = n$ vertices, then there are $n!$ such isomorphisms, together forming the *symmetric group* S_n on n elements. The *isomorphism class* of G is the set of labeled graphs

$$\{\alpha G : \alpha \in S_n\}.$$

Note that an isomorphism class might not have size $n!$ since it is possible that $G = \alpha G$ for some non-trivial α.

There are two common ways to represent an isomorphism class of graphs. Firstly, a graph is called *unlabeled* if its vertices are indistinguishable (except through their adjacencies). In this case, we omit labels when drawing the graph. Assigning vertex labels to an unlabeled graph in all possible ways yields the whole isomorphism class.

An example of an unlabeled graph and its corresponding isomorphism class is given below.

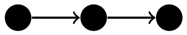

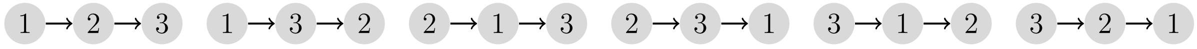

The second way is by choosing a representative element of the isomorphism class. Let Γ be a set of labeled graphs, e.g. the set of k-node labeled directed graphs. Then a function C whose domain is Γ is called a *canonical labeling* if $C(G) = C(G')$ for $G, G' \in \Gamma$ if and only if G and G' are isomorphic. The *canonical label* of a graph G therefore determines which isomorphism class it belongs to. Simply put, if we input two isomorphic graphs, we get the same output, and if we input two non-isomorphic graphs, we get different outputs.

In NetMODE, we take the canonical label approach. We will now give the details of our canonical labeling. An *adjacency matrix* of a graph $G = (V, E)$ on $|V| = n$ nodes is an $n \times n$ matrix whose (i, j)-th entry is 1 if $(i, j) \in E(G)$ and 0 otherwise. From the adjacency matrix we can obtain a binary number by reading from left-to-right and top-to-bottom. We can convert this to a decimal number, which we call the *graphID*. Therefore every number from 0 to $2^{n^2} - 1$ is the graphID of a labeled digraph on n vertices (although, some will have loops). We define the canonical label $C(G)$ to be the minimum graphID amongst all labeled graphs in the isomorphism class containing G. In the example below, the canonical label would be 56044064, which can be found be computing the graphID of every isomorphic graph (there are 720 of them).

Since NetMODE ignores loops, the adjacency matrix of all graphs is assumed to have an all-0 main diagonal, so to save space, NetMODE discards this redundant information. Every graph therefore has an *internal graphID*, formed in the same way as the normal graphID, but the main diagonal is skipped.

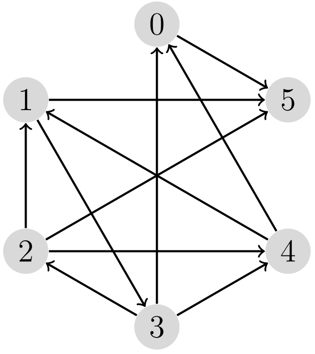

0	0	0	0	0	1
0	0	0	1	0	1
0	1	0	0	1	1
1	0	1	0	1	0
1	1	0	0	0	0
0	0	0	0	0	0

100010101001110101011000000000   1162783744

adjacency matrix;   in binary;   graphID

Keep in mind that the number of nodes of the graph is somehow implicit in this type of canonical label. To illustrate, the k-node null graph (which has k nodes and no edges) has the canonical label 0, regardless of k. For the purpose of network motif detection, this is not a problem since we consistently stick to a fixed k.

1.2 The Reconstruction Conjecture

In this section we deal exclusively with unlabeled graphs. Given a graph $G = (V, E)$ on $|V| = n$ vertices, we can construct another graph from G by deleting one of the vertices $v \in V$ along with the edges which have v as an endpoint; this graph is denoted $G \setminus v$. The multiset $\{G \setminus v : v \in V\}$ is called the *deck* of G and any element in the deck of G is called a *card*. Note that a deck is a multiset, i.e. it can contain multiple copies of the same element.

The Reconstruction Conjecture states that, for undirected graphs, if two graphs on three or more vertices have the same deck, then they are isomorphic [4].

However, for the purposes of NetMODE, we are interested in the variant of the Reconstruction Conjecture for loop-free directed graphs. In fact, it has been known for some time that there are so-called nonreconstructable digraphs [3, 19, 20], that is, the Reconstruction Conjecture is false for digraphs. However, for small k at least, there are very few exceptions to the Reconstruction Conjecture for digraphs. For $k = 6$, we find exactly nine exceptions, which are tabulated below using their canonical labels.

deck						G	G'
43798	43828	44816	108176	123412	124432	56044064	56036128
51088	116368	1090436	1163908	1165830	1165860	1133217864	1133217802
549790	1598350	1598350	1631110	1631110	1631110	1640468230	1640206094
582550	1598380	1599370	1599370	1663630	1663660	1657244500	1656986394
582580	1598350	1599370	1599370	1631110	1663660	1657252436	1656990300
1126296	1158036	1166094	1166124	1167124	3253900	3311111272	3311111210
1599370	1631110	1663630	1663660	1663660	3727750	3788211532	3788211470
1663918	1664926	1664956	1666732	3729822	3730842	3838293818	3838289726
1666970	1698710	3728810	3728820	3731244	3794604	3856371052	3856370990

The graph 1162783744, drawn on page 4, has the canonical label 56044064, and by replacing the edge $(3, 2)$ with $(2, 3)$, we obtain a graph with the canonical label 56036128 (i.e. these are the two 6-node graphs in the first exception).

1.2.1 Application in NetMODE

A list of all 5-node loop-free graphs can easily be stored in memory; it can consist of 2^{20} `int`s (32-bit). In the 6-node case, we instead have 2^{30} graphs, which is too many to be practically stored in memory. As such, we use the Reconstruction Conjecture for digraphs to essentially change the canonical label for 6-node graphs to their decks of 5-node subgraphs (barring the few exceptions).

We need to quickly determine whether or not a subgraph H is one of the exceptions listed above. This is achieved according to Algorithm 1.

Algorithm 1 Is H an exceptional subgraph?

int $t := H \pmod{7045650}$.
if $H =$ `exception_table`$[t]$ **then**
  H is an exception.
  Increase `exceptionID`$[t]$.
else
  H is not an exception (so continue as normal).
end if

During the preprocessing phase, we generate and store an array `exception_table` of length 7045650. This array is designed so that if H is a connected 6-node subgraph and `exception_table`$[t] = H$, where $t = H \mod 7045650$, then H is an exceptional subgraph, and we instead increase the value of `exceptionID`$[t]$, an array which stores the counts of the exceptional subgraphs. The vast majority of the elements in `exception_table` are 0.

1.3 Similar graphs

If v is a vertex in a graph $G = (V, E)$, then its *in-degree* is the number of edges of the form $(i, v) \in E$. Similarly its *out-degree* is the number of edges of the form $(v, j) \in E$.

Given an input network $G = (V, E)$, a *similar* graph is defined to be a graph with the same vertices V and for which each $v \in V$ has the same in-degrees and out-degrees. There are some problems with this definition:

Q1 How can we practically generate similar graphs?

Q2 How are bidirectional edges treated?

1.3.1 Kavosh and FanMod

The answer to the first question (Q1) was addressed in Kavosh and FanMod (among other programs) by a switching method, similar to the method described in [15]. The switching operation is quite simple: two edges (a, c) and (b, d) are chosen, and are replaced by (a, d) and (b, c) provided no loops or multiple edges are introduced.

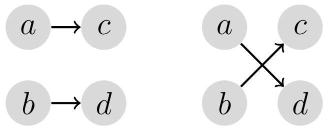

Given a vertex $v \in V$ in a graph $G = (V, E)$, the *neighborhood*, denoted $N(v)$, of v is the set of vertices i for which either $(i, v) \in E$ or $(v, i) \in E$ (or both). Vertices in $N(v)$ are called the *neighbors* of v. The *out-neighborhood*, denoted $N_{\text{out}}(v)$, is the set of vertices i for which $(v, i) \in E$. Vertices in $N_{\text{out}}(v)$ are called the *out-neighbors* of v.

Rather than uniformly choose the two edges in the switch, Kavosh iterates through each vertex a several times. Once a is chosen, Kavosh then picks another vertex b at random ($a \neq b$), and chooses random out-neighbors $c \in N_{\text{out}}(a)$ and $d \in N_{\text{out}}(b)$. Kavosh will apply the switch provided the following conditions are satisfied:

- $(a, c) \in E$,
- $c \neq b$,
- $(b, d) \in E$,
- $d \neq a$,
- $(b, c) \notin E$,
- $(c, a) \notin E$,
- $(a, d) \notin E$,
- $(d, b) \notin E$.

Consequently, Kavosh will also leave all bidirectional edges fixed, that is, if both (u, v) and (v, u) are present in the input network, then every subsequent comparison graph will have both (u, v) and (v, u). This can result in some undesirable behavior. For example, regardless of the input network, Kavosh would never declare as a motif e.g. the complete directed graph $\vec{K}_3$, since it will occur no fewer times in the input graph than in any comparison graph. However, $\vec{K}_3$ (also known as the "fully connected triad") was considered a motif in a world wide web network in [16]. Furthermore, it is possible to satisfy Kavosh's conditions while e.g. $(d, a) \in E$, in which case, after the switch is performed, a bidirectional edge between a and d is created, and will remain for each subsequent comparison graph generated.

Importantly, the switches applied by Kavosh are not always reversible. This can result in misleading significance levels. Figure 6 (on page 22) compares the Z-scores returned of Kavosh, FanMod, and NetMODE, running the same experiment. Aside from "no regard" (NR) mode (which is not a suitable switching method in this case), we see that Kavosh's results are (a) significantly larger than the other results and (b) vary considerably more.

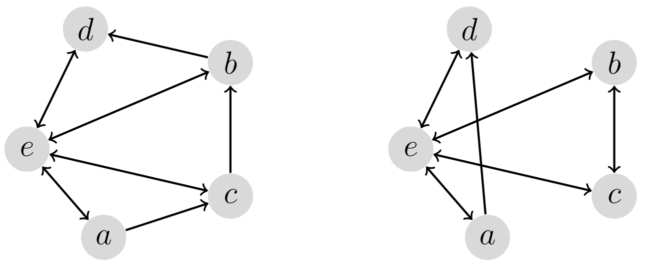

Figure 1: Kavosh's switching method will, at some point, change the left graph into the right graph, which subsequently will never be modified.

The reader can verify the problem with Kavosh's switching method by running Kavosh on the graph on the left hand side in Figure 1, where only one possible switch can be performed by Kavosh. Eventually, Kavosh will make this switch, thereby generating a bidirectional edge which will never be modified. This effect would be apparent from Kavosh's results, however, it is obscured by two bugs: (a) the IDs are mislabeled and (b) to compute the concentrations in the comparison graphs, Kavosh incorrectly divides by the total number of graphs in the input network (when it should be the total number of graphs in the corresponding comparison graph).

Note that all of the bugs we have found in Kavosh could be easily repaired.

FanMod, on the other hand, allows the user to choose one of three different ways of handling bidirectional edges [22].

1. *No regard.* Bidirectional edges are not preserved.

2. *Global constant.* The total number of bidirectional edges is fixed.

3. *Local constant.* The number of adjoining bidirectional edges at each vertex is preserved.

1.4 Configuration Model

We will now introduce the version of the Configuration Model used in NetMODE, and verify its correctness. We refer the reader to [17] for more details on the Configuration Model. We call this algorithm the *Mixed Graph Configuration Model.* We will consider a *mixed graph* as a graph that can have either single-directional or undirected edges between nodes (and no parallel edges). There are other definitions of "mixed graph" around. A digraph can be interpreted as a mixed graph by replacing each bidirectional edge with an undirected edge, and by our definition, the converse holds too. While we will not deal with mixed graphs directly, we will treat bidirectional edges as distinct from single-directional edges, thereby motivating the title "Mixed Graph Configuration Model".

In "local constant" mode, we define a graph as *similar* to G if it (a) has the same vertices, (b) each vertex has the same in-degree and out-degree as G, and (c) each vertex has the same bidirectional degree (i.e. is the endpoint of the same number of bidirectional edges as in G).

The Mixed Graph Configuration Model proceeds as follows.

Stage 1. Suppose $\{v_{2i-1}, v_{2i}\}_{1\leq i\leq k}$ is a list of bidirectional edges in G. Let α be a permutation of $\{1, 2, \ldots, 2k\}$ chosen uniformly at random. Then the candidate edges for the random graph are $\{v_{\alpha(2i-1)}, v_{\alpha(2i)}\}_{1\leq i\leq k}$. If these candidates contain a loop or parallel edge, we restart the algorithm.

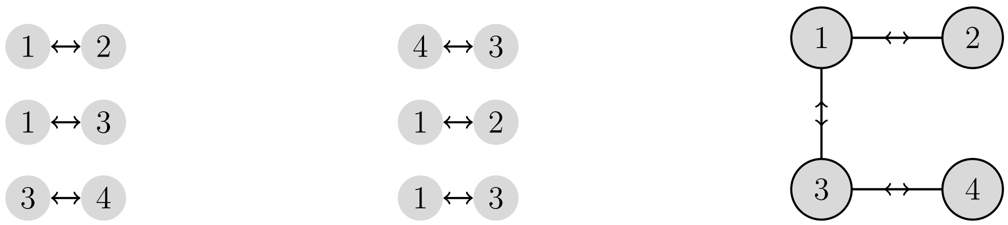

Figure 2: Illustrating Stage 1 of the Mixed Graph Configuration Model. The graph on the left hand side lists the bidirectional edges. After randomly permuting the labels, we obtain the middle graph. The graph on the right hand side is generated from the graph in the middle by identifying vertices.

Stage 2. At this stage, we collect the list of single-directional edges, which we label as $(x_i, y_i)_{1\leq i\leq k}$. Let β be a permutation of $\{1, 2, \ldots, k\}$ chosen uniformly at random. Then the candidate edges for the random graph are $(x_i, y_{\beta(i)})_{1\leq i\leq k}$. If these candidates edges contain a loop, parallel edge (including the candidates at Stage 1), or bidirectional edge, we restart the algorithm.

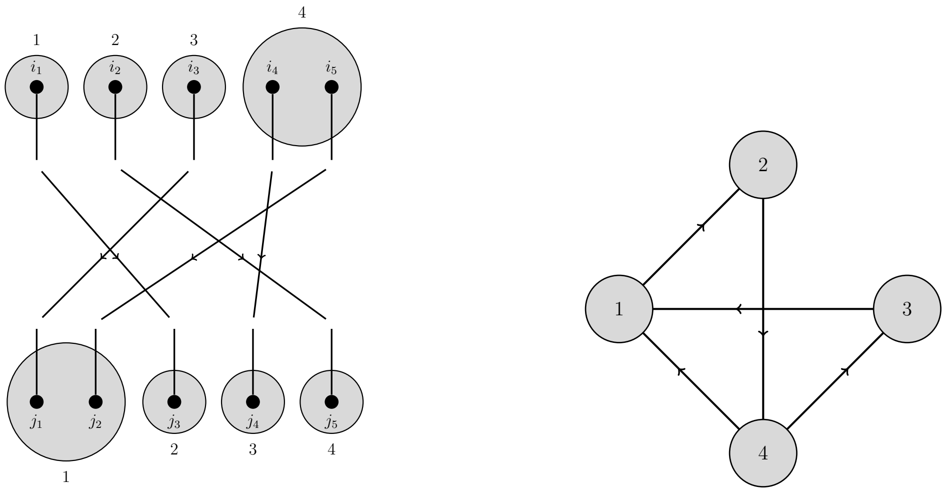

Figure 3: Illustrating Stage 2 of the Mixed Graph Configuration Model. The graph on the left hand side is generated from a random permutation. The graph on the right hand side is generated from the graph on the left by identifying vertices.

Return graph. We return the graph with bidirectional edges $\{v_{\alpha(2i-1)}, v_{\alpha(2i)}\}_{1\leq i\leq k}$ and single-directional edges $(x_i, y_{\beta(i)})_{1\leq i\leq k}$.

Note that generating random permutations uniformly at random can be efficiently achieved by using a Fisher-Yates Shuffle. Note also that it is important to restart from scratch whenever a clash arises. If, for example, we restarted only Stage 2 when a Stage 2 clash arises, then the resulting distribution would be non-uniform (since the probability of restarting Stage 2 could vary with the results of Stage 1).

1.4.1 Uniformity

We will now show that the Mixed Graph Configuration Model indeed generates similar graphs uniformly at random.

Let out(v) and in(v) respectively denote the out-degree and in-degree of vertex v in G (including only singly directed edges). Let deg(v) denote the degree of vertex v (including only bidirectional edges).

Theorem 1. *Let H be a graph similar to the input network G. Then the probability that the Mixed Graph Configuration Model generates H is*

$$\Big(\frac{2^r r! \prod_v \deg(v)!}{(2r)!}\Big) \cdot \Big(\frac{\prod_v \mathrm{in}(v)!\mathrm{out}(v)!}{s!}\Big),$$

where r is the number of bidirectional edges in G and s is the number of single-directional edges in G. Therefore, the Mixed Graph Configuration Model generates random graphs uniformly at random.

Proof. This will be largely a "proof by example" designed to illustrate the concepts behind the proof (aimed at a non-mathematical audience). An actual proof readily follows from the theory of group actions, in particular, the Orbit-Stabilizer Theorem.

Firstly, we observe that, since H and G are similar graphs, r is the number of bidirectional edges in H and s is the number of single-directional edges in H.

Stage 1. We will begin with an illustrative example. Consider the case when G has the bidirectional edges: $\{1,2\},\{1,3\},\{1,4\},\{2,4\},\{2,5\}$. From this set, we construct a list $\ell = (1,2,1,3,1,4,2,4,2,5)$. We can imagine that the algorithm would randomly permute ℓ (using α) then return a list of candidate bidirectional edges from that list. For example, if $(2,1,1,5,1,4,2,4,2,3)$ was obtained by the random permutation, then the candidates would be $\{2,1\},\{1,5\},\{1,4\},\{2,4\},\{2,3\}$. This is a *valid* list of candidates since it contains no loops nor parallel edges. However, the order in which the candidates are listed does not matter, nor does the order in which the elements in each candidate is listed (since the edges are bidirectional). So, of all possible distinct lists formed by permuting the elements of ℓ, we find exactly $2^5 5!$ of them would give rise to the above set of valid candidates. In fact, this is true in general; the number of distinct lists that give rise to a valid set of candidates is $2^r r!$. (They are all distinct since a valid list of candidates must not have loops or parallel edges.)

There is also a second point to raise. There are permutations α that do not give distinct lists. For example, if we swap two of the 1's in ℓ, we obtain ℓ back again. In fact, we can get ℓ back again via $3! \cdot 3! \cdot 1! \cdot 2! \cdot 1! = 72$ different permutations. Moreover, of the 10! lists that are obtained by permuting ℓ, we will find exactly 72 copies of every list. In general,

we will find $\prod_v \deg(v)!$ copies of every list (since $\deg(v)$ is the number of copies of v in the list). We invoke the Orbit-Stabilizer Theorem here.

We conclude that there are exactly $2^r r! \prod_v \deg(v)!$ permutations α that give rise to any given valid list of candidate edges. Hence the probability that a given valid list of candidates (and thus the bidirectional component of H) is returned after one iteration of Stage 1 is

$$\frac{2^r r! \prod_v \deg(v)!}{(2r)!}.$$

Stage 2. Again we will begin with an example. Consider the case when G has the singly directed edges: $(1,5),(2,5),(2,6),(5,3),(5,6),(5,7)$. We construct two lists $x_G = (1,2,2,5,5,5)$ and $y_G = (5,5,6,3,6,7)$ from the elements in the first and second coordinates, respectively. This time, we randomly permute y_G (using β) and return the pairs $(x_G[i], y_G[\beta(i)])$ as the candidate edges.

In the same vein as with G, the similar graph H comes with a set of singly directed edges, that also gives rise to two lists x_H and y_H. In fact, we can assume $x_H = x_G$ (otherwise, we can re-label H so that $x_H = x_G$, and if this is impossible then H and G are not similar). We also know that y_H and y_G have the same elements, and the same number of copies of each element (again, otherwise H and G are not similar). So, it is possible to permute y_G to obtain y_H.

The number of ways of permuting y_H (or y_G, or, in fact, any list with the same multiset of symbols as y_H) to obtain y_H back again is $\prod_v \mathrm{in}(v)!$. Additionally, there are $\prod_v \mathrm{out}(v)!$ ways of permuting x_G (or x_H) to obtain x_G back again, or equivalently, there are $\prod_v \mathrm{out}(v)!$ distinct lists formed by permuting y_H that would give rise to the list of candidates that match H (since there are no loops or parallel edges). We also invoke the Orbit-Stabilizer Theorem here. We can therefore conclude that there are exactly $\prod_v \mathrm{in}(v)! \cdot \mathrm{out}(v)!$ permutations β in which we would obtain the same set of candidate edges as in y_H.

Conclusion. The event "Stage 2 agrees with H" is independent of the event "Stage 1 agrees with H". Thus the probability that H is returned by a single iteration of the Mixed Graph Configuration Model is

$$\begin{aligned}\Pr(H \text{ returned}) &= \Pr(\text{Stage 1 agrees with } H \text{ and Stage 2 agrees with } H)\\ &= \Pr(\text{Stage 1 agrees with } H) \times \Pr(\text{Stage 2 agrees with } H)\\ &= \Big(\frac{2^r r! \prod_v \deg(v)!}{(2r)!}\Big)\Big(\frac{\prod_v \mathrm{in}(v)! \cdot \mathrm{out}(v)!}{s!}\Big).\end{aligned}$$

□

Note that uniformity is not affected by which stage we actually perform first, provided we restart from scratch whenever a clash arises. In some cases, it is faster to perform Stage 2 first so NetMODE chooses between these two options.

1.4.2 Comparison graphs in NetMODE

For the switching methods in NetMODE we retain Kavosh's method for choosing pairs of edges, while allowing the user to choose one of the three switching modes, similar to that

in FanMod, along with a Kavosh-like switching mode, and the Mixed Graph Configuration Model. The following options are therefore possible:

0. *Fixed.* Bidirectional edges are fixed in place.
1. *No regard.* Bidirectional edges are not preserved.
2. *Global constant.* The total number of bidirectional edges is fixed.
3. *Local constant.* The number of adjoining bidirectional edges at each vertex is preserved.
4. *Uniform local constant.* Local constant mode, uniformly at random via the Mixed Graph Configuration Model.

The user should decide for themselves which mode to use. For example, if the user's network was derived from a source in which bidirectional edges are impossible (e.g. a family tree), local constant (or global constant) would likely give a better comparison (N.B. "no regard" mode may create bidirectional edges).

In Figure 4 we tabulate the possible edge configurations that can be modified by NetMODE. The left hand side of the table indicates the edges between a, b and c, d, while the top of the table indicates the edges between a, c and b, d. The corresponding cell shows the resulting subgraph after the switch has been performed and indicates under which settings that switch will be performed. The edges between a and b and between c and d (or lack thereof) do not affect the switching process and are not taken into account. Configurations that contain an edge (a, d) or (b, c) would give rise to a parallel edge, so these switches are forbidden and consequently omitted from Figure 4.

2 Using NetMODE

2.1 R

We have provided a basic script that enables calling of NetMODE from R (using the igraph library). To use it, the following line needs to be edited in NetMODER.txt:

```
NetMODECommand <- "c:/users/dougy/dropbox/NetMODE/NetMODE.exe"
```

to correspond to the user's NetMODE executable.

Once this is done, assuming igraph is already installed, NetMODE can be initialized by the following command in R:

```
source("c:/users/dougy/dropbox/NetMODE/NetMODER.txt")
```

where again the directory will need to be changed. After which, the user should be able to call NetMODE from R, such as via:

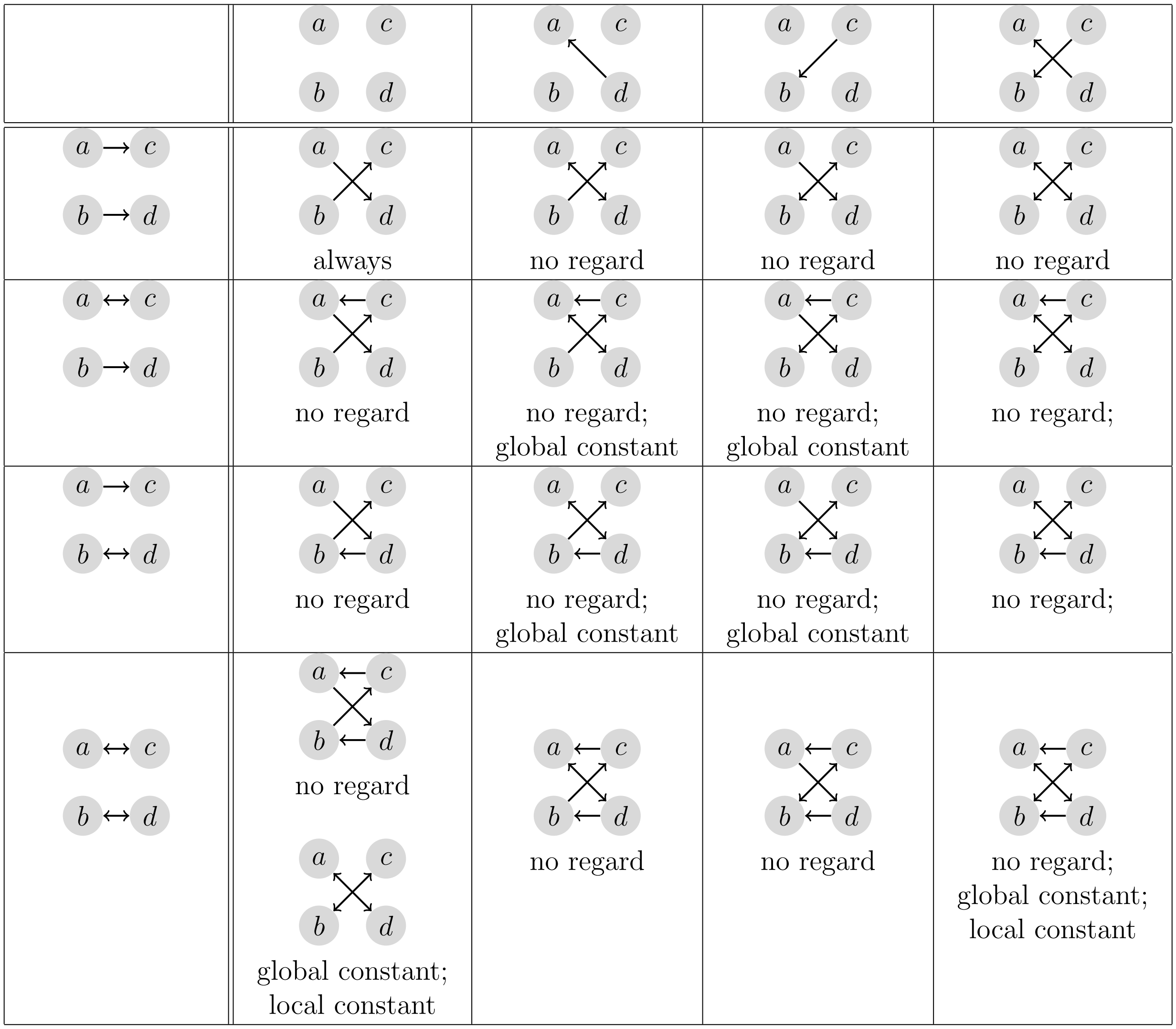

Figure 4: The 16 possible edge configurations that can be modified by NetMODE.

```
> g <- KavoshToIGraph("c:/users/dougy/dropbox/NetMODE/networks/ecoli")
Read 2553 items
> z <- NetMODE(g,3,10,0,1,1)
> z
[[1]]
 [1]   6  12  14  36  38  46  74  78  98 102 110 238

[[2]]
 [1] 196 322 553 167  12   1 536 758   2   3  17  23

[[3]]
[[3]][[1]]
 [1] 1379 1480 1556 1589 1589 1512 1573 1583 1583 1537

[[3]][[2]]
 [1] 3451 3672 3821 3901 3876 3702 3854 3875 3866 3768

[[3]][[3]]
 [1] 218 122  54  18  21  91  29  31  30  72
```

The remaining lines have been snipped for brevity. R calls NetMODE in "verbose" mode, where all of the intermediate data is returned. The data stored in `z` is defined below.

- `z[[1]][j]` contains the GraphID of the `j`-th graph in `g`. We can look at this graph with:

  ```
  > GraphIDToGraph(3,z[[1]][2])
  Vertices: 3
  Edges: 2
  Directed: TRUE
  Edges:

  [0] 2 -> 0
  [1] 1 -> 2
  > GraphIDToAdjacencyMatrix(3,z[[1]][2])
       [,1] [,2] [,3]
  [1,]    0    0    0
  [2,]    0    0    1
  [3,]    1    0    0
  > m <- GraphIDToGraph(3,z[[1]][2])
  > plot(m,layout=layout.fruchterman.reingold,vertex.size=40)
  ```

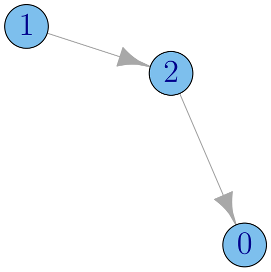

- `z[[2]][j]` contains the number of copies (up to isomorphism) of the `j`-th graph in `g`.
- `z[[3]][[j]][i]` contains the number of copies of the `j`-th graph in `g` in the `i`-th comparison graph.

We can check whether or not a normal approximation is appropriate visually via:

```
> z <- NetMODE(g,3,10000,0,1,1)
> t <- z[[3]][[2]]
> plot(table(t))
```

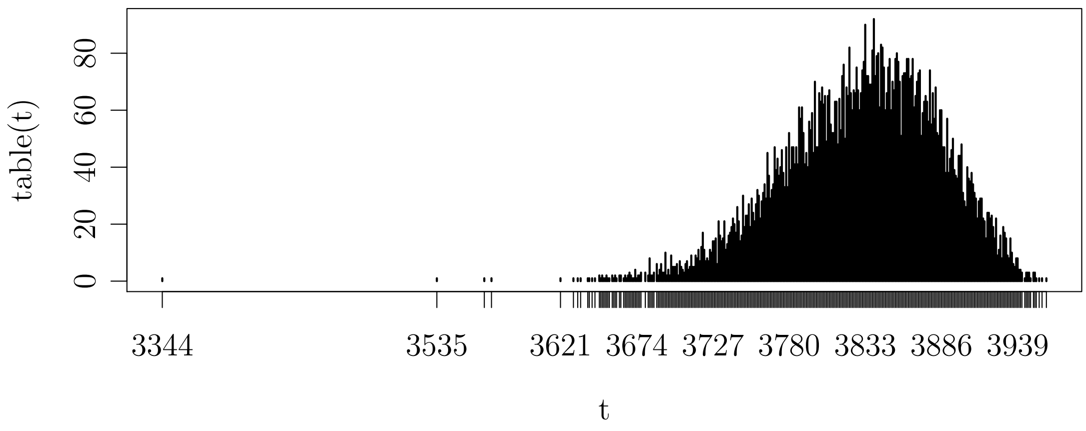

In this instance `z[[2]][2]`= 322, which is surprisingly small. We could also perform a normality test, e.g.:

```
> shapiro.test(t[1:5000])

        Shapiro-Wilk normality test

data:  t[1:5000]
W = 0.8498, p-value < 2.2e-16
```

The theory of network motifs has been subject to some criticism; for example, see [2, 6, 7, 8, 9, 11] (see also [14]). NetMODE's R extension allows the user to perform their own statistical analyses. It returns all of the subgraph counts, so can return a lot of data.

2.2 Command line

NetMODE runs much faster via the command line. A typical instance is called via e.g.:

```
NetMODE -k 3 -c 1000 -b 0 -e 3 -t 1 < ./networks/ecoli > output.txt
```

There parameters are defined by:

- We are performing a k-node subgraph census, where k is restricted to the range $3 \leq k \leq 6$.
- There are c comparison graphs.
- There is b burnin used.
- The edge switching method used is 3: local constant switching mode (see page 11 for the complete list of methods possible).
- We limit NetMODE to use t threads simultaneously.
- The input network is `./networks/ecoli`. This input network, along with other examples are provided with NetMODE.
- The output is written to `output.txt`.
- Note that "verbose mode", invoked with `-v 1`, is intended to be used for interfacing with R (or some user-built package).

A typical line in `output.txt` would begin:

```
gID:  46  freq:     1  ave_rand_freq:     0.31 (sd:  0.569)
```

The `gID` is the graphID of the k-node subgraph found in the input network (in this case 46). The frequency is given by `freq` (in this case 1). The `ave_rand_freq` is the average frequency of this subgraph in the ensemble of random similar graphs, and `sd` is its emperical standard deviation. This line continues:

```
conc: 0.00039  ave_rand_conc: 0.00012 (sd: 0.0002114)
```

which gives the concentration-based statistics. The concentration of this subgraph is given by `conc` (in this case 0.00039). The `ave_rand_conc` is the average concentration of this subgraph in the ensemble of random similar graphs, and `sd` is its emperical standard deviation. This line continues:

```
f-ZScore:   1.21  f-pValue: 0.26100000  c-ZScore:   1.28  c-pValue: 0.04500000
```

which gives measures of statistical significance: (a) the frequency-based Z-score `f-ZScore`, (b) the frequency-based p-value `f-pValue`, (c) the concentration-based Z-score `c-ZScore`, and (d) the concentration-based p-value `c-pValue`. Note that these four results might be contradictory, so the user should be careful when declaring which subgraphs are motifs.

The user can also invoke the switch `-s` which ensures that a line of data is returned for every k-node connected subgraph encountered, regardless of whether it is an induced subgraph of the input network. This switch only works when $k \leq 5$, due to the structure of NetMODE.

The switch `-k` must be set. The remainder are optional. If they are omitted, the defaults are 0 comparison graphs, 0 burnin, edge switching method 3 (local constant mode), and 1 thread.

3 Further testing of NetMODE

3.1 Number of comparison graphs

Figure 5 (on page 21) plots the run-time by NetMODE as the number of comparison graphs varies. In Figure 5 we perform (a) a 5-node subgraph census on the E. Coli dataset, (b) a 6-node subgraph census on the E. Coli dataset, and (c) a 6-node subgraph census on the social network. Figure 5 shows that, after the initial hump due to the overhead associated with preprocessing, NetMODE achieves constant speedup vs. Kavosh as the number of comparison graph increases.

3.2 E. Coli transcription factor network

We ran NetMODE on the E. Coli transcription factor network downloadable from the RegulonDB website [5].

The feedforward loop is clearly overrepresented in the network (settings: `-k 3 -c 10000 -b 0 -e 3`):

```
gID:  38  freq:  1141  ave_rand_freq:   574.48 (sd: 55.715)
conc: 0.00403  ave_rand_conc: 0.00202 (sd: 0.0001966)
f-ZScore:  10.17  f-pValue: 0.00000000  c-ZScore:  10.25  c-pValue: 0.00000000
```

as is the bifan (settings: `-k 4 -c 1000 -b 0 -e 3`):

```
gID:   204  freq: 43741  ave_rand_freq: 28424.20 (sd: 2982.713)
conc: 0.00167  ave_rand_conc: 0.00107 (sd: 0.0001139)
f-ZScore:    5.14  f-pValue: 0.00000000  c-ZScore:    5.23  c-pValue: 0.00000000
```

both of which were detected as network motifs by Milo et al. [16]

In the case of 5-node motifs, the most significant results are given below (settings: `-c 10000 -b 0 -e 3`). We include only cases where both the frequency and concentration p-values are at most 0.0001. We exclude cases with low frequencies (all of the motifs occur more than 1000 times). During the computation, around 2×10^{13} subgraphs were encountered, and 84% of these graphs belong to the same isomorphism class.

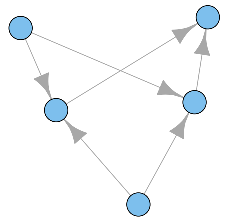

gID = 101904
frequency Z-score = 77.56

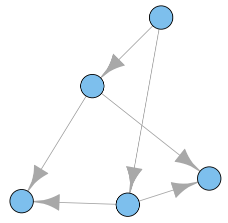

gID = 3864
frequency Z-score = 46.99

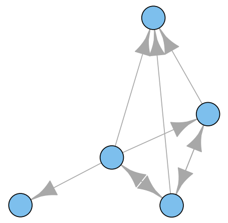

gID = 10158
frequency Z-score = 39.13

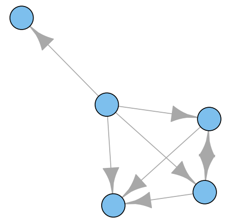

gID = 10156
frequency Z-score = 27.60

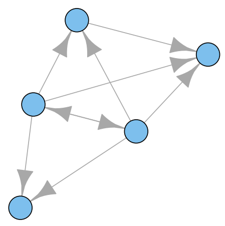

gID = 9150
frequency Z-score = 19.41

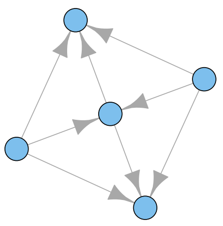

gID = 25500
frequency Z-score = 17.18

3.3 Output comparison with Kavosh and FanMod

Figure 6 (on page 22) lists the Z-score returned by Kavosh, FanMod, and NetMODE on the E. Coli network. In each case we look for the 4-node graph

$$\bullet\longleftrightarrow\bullet\longleftrightarrow\bullet\longleftrightarrow\bullet.$$

It has graphID 4698 (minimum) or 8598 (as returned by Nauty), and each experiment was run three times to get a feeling for the level of consistency. The number of comparison networks was $N \in \{100, 1000, 10000\}$. For NetMODE, the burnin was taken from $\{0, 10000\}$. Figure 6 uses the notation: F = fixed bidirectional edges (`-e 0`); NR = no regard (`-e 1`); GC = global constant (`-e 2`); LC = local constant (`-e 3`); ULC = uniform local constant (`-e 4`); f = frequency Z-score; c = concentration Z-score; b = burnin applied (`-b 10000`).

We make the following observations:

- The LC and ULC values returned by NetMODE are quite close, suggesting that the switching method in LC mode is reasonably close to uniform. Also, the results of FanMod's LC mode differ considerably from NetMODE's ULC.
- NetMODE's GC mode seems to benefit from the use of burnin.

3.3.1 Bias in FanMod

Figure 6, along with the results in the main text, highlight a built-in bias in FanMod. We will now give some more experimental results that indicate the extend to which this bias applies.

If we repeat the experiment in the main text with 100000 comparison graphs. We instead obtain Z-scores of -43.424 and 87.669 in FanMod LC and -16.59 and 19.21 in NetMODE ULC, for the subgraphs with graphID 78 and 238, respectively (E. Coli network, 3-node subgraph census). So the bias is not mitigated by repeating the experiment nor increasing the number of comparison graphs.

For $k = 6$, E. Coli network:

- The subgraph with graphID 1092133754 (or 2182389326) returned a Z-score of 6084.6 in FanMod, 47.29 in NetMODE LC, and 57.44 in NetMODE ULC.
- The subgraph with graphID 1091318638 (or 5386965102) returned a Z-score of 3512.5 in FanMod, 10.61 in NetMODE LC, and 10.97 in NetMODE ULC.

Both of these subgraphs have a single-directional edge. This also shows that FanMod's bias is not limited to the $k = 3$ case, and can have a huge effect. If fact, this bias is so large that it seems that the most significant results found by FanMod are simply the result of this bias.

We can also observe similar behaviour for undirected input networks, as indicated in Table 7 (on page 22). The graph used in this experiment is the western states power grid of the USA [21] sourced from `http://www-personal.umich.edu/~mejn/netdata/`.

References

[1] N. Alon, P. Dao, I. Hajirasouliha, F. Hormozdiari, and S. C. Sahinalp, *Biomolecular network motif counting and discovery by color coding*, Bioinformatics, 24 (2008).

[2] Y. Artzy-Randrup, S. J. Fleishman, N. Ben-Tal, and L. Stone, *Comment on "Network motifs: Simple building blocks of complex networks" and "Superfamilies of evolved and designed networks"*, Science, 305 (2004), p. 1107.

[3] L. W. Beineke and E. T. Parker, *On nonreconstructable tournaments*, Journal of Combinatorial Theory, 9 (1970), pp. 324–326.

[4] J. A. Bondy and R. L. Hemminger, *Graph reconstruction – a survey*, J. Graph Theory, 1 (1977), pp. 227–268.

[5] S. Gama-Castro, H. Salgado, M. Peralta-Gil, A. Santos-Zavaleta, L. Muniz-Rascado, H. Solano-Lira, V. Jimenez-Jacinto, V. Weiss, J. S. Garcia-Sotelo, A. Lopez-Fuentes, L. Porron-Sotelo, S. Alquicira-Hernandez, A. Medina-Rivera, I. Martinez-Flores, K. Alquicira-Hernandez, R. Martinez-Adame, C. Bonavides-Martinez, J. Miranda-Rios, A. M. Huerta, A. Mendoza-Vargas, L. Collado-Torres, B. Taboada, L. Vega-Alvarado, M. Olvera, L. Olvera, R. Grande, E. Morett, and J. Collado-Vides, *RegulonDB version 7.0: transcriptional regulation of Escherichia coli K-12 integrated within genetic sensory response units (gensor units)*, Nucleic Acids Res., 39 (2010), pp. D98–D105.

[6] B. Goemann, E. Wingender, and A. P. Potapov, *An approach to evaluate the topological significance of motifs and other patterns in regulatory networks*, BMC Systems Biology, 3 (2009).

[7] P. J. Ingram, M. P. Stumpf, and S. J., *Network motifs: structure does not determine function*, BMC Genomics, 7 (2006).

[8] J. F. Knabe, C. L. Nehaniv, and M. J. Schilstra, *Do motifs reflect evolved function? - no convergent evolution of genetic regulatory network subgraph topologies*, BioSystems, 94 (2008), pp. 68–74.

[9] A. S. Konagurthu and A. M. Lesk, *On the origin of distribution patterns of motifs in biological networks*, BMC Systems Biology, 2 (2008).

[10] M. Koskas, G. Grasseau, E. Birmelé, S. Schbath, and S. Robin, *NeMo: Fast count of network motifs*, in Book of Abstracts for Journées Ouvertes Biologie Informatique Mathématiques (JOBIM) 2011, 2011, pp. 53–60.

[11] D. Marbach, R. J. Prill, T. Schaffter, C. Mattiussi, D. Floreano, and G. Stolovitzky, *Revealing strengths and weaknesses of methods for gene network inference*, Proc. Natl. Acad. Sci. USA, 107 (2010), pp. 6286–6291.

[12] D. Marcus and Y. Shavitt, *Efficient counting of network motifs*, in Proc. 30-th International Conference on Distributed Computing Systems Workshops, Conference Publishing Services, 2010, pp. 92–98.

[13] ——, *RAGE - a rapid graphlet enumerator for large networks*, Computer Networks, (2011). To appear.

[14] R. Milo, S. Itzkovitz, N. Kashtan, R. Levitt, and U. Alon, *Response to comment on "Network motifs: Simple building blocks of complex networks" and "Superfamilies of evolved and designed networks"*, Science, 305 (2004), p. 1107.

[15] R. Milo, N. Kashtan, S. Itzkovitz, M. E. J. Newman, and U. Alon, *On the uniform generation of random graphs with prescribed degree sequences*, (2003). arXiv:cond-mat/0312028v2 [cond-mat.stat-mech].

[16] R. Milo, S. Shen-Orr, S. Itzkovitz, N. Kashtan, D. Chklovskii, and U. Alon, *Network motifs: Simple building blocks of complex networks*, Science, 298 (2002), pp. 824–827.

[17] M. Newman, *Networks: An Introduction*, Oxford University Press, 2010.

[18] S. Omidi, F. Schreiber, and A. Masoudi-Nejad, *MODA: An efficient algorithm for network motif discovery in biological networks*, Genes Genet. Syst., 84 (2009), pp. 385–395.

[19] P. K. Stockmeyer, *The falsity of the reconstruction conjecture for tournaments*, Journal of Graph Theory, 1 (1977), pp. 19–25.

[20] ——, *A census of non-reconstructable digraphs, I: Six related families*, Journal of Combinatorial Theory, Series B, 31 (1981), pp. 232–239.

[21] D. J. Watts and S. H. Strogatz, *Collective dynamics of "small-world" networks.*, Nature, (1998), pp. 440–442.

[22] S. Wernicke and F. Rasche, *FANMOD: a tool for fast network motif detection*, Bioinformatics, 22 (2006), pp. 1152–1153.

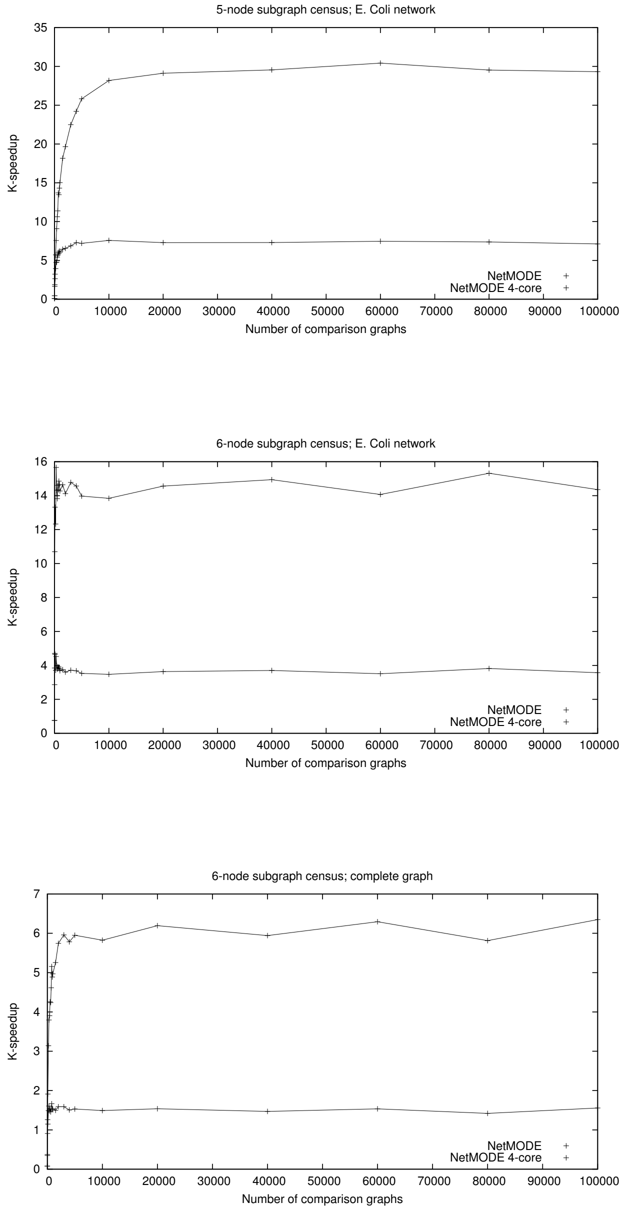

Figure 5: Speedup of NetMODE vs. Kavosh (K-speedup) as the number of comparison graphs varies.

N	Kavosh			FanMod, NR			FanMod, GC			FanMod, LC		
100	16.65	421.10	687.94	51612	10130	36047	3.02	3.21	3.08	1.78	1.61	1.91
1000	614.84	58.68	635.67	28861	33316	22808	2.99	3.01	3.02	1.76	1.75	1.78
10000	929.29	387.14	270.75	28524	25104	28765	3.02	3.01	2.97	1.73	1.75	1.73

N	NetMODE, Ff			NetMODE, Fc			NetMODE, Ffb			NetMODE, Fcb		
100	−4.39	−3.75	−5.26	2.90	2.12	2.01	−6.90	−3.17	−3.30	2.18	2.82	2.22
1000	−3.78	−3.52	−3.82	2.23	2.24	2.23	−3.63	−3.88	−3.65	2.18	2.16	2.32
10000	−3.92	−3.63	−3.55	2.22	2.20	2.21	−3.97	−3.81	−3.96	2.20	2.20	2.19

N	NetMODE, NRf			NetMODE, NRc			NetMODE, NRfb			NetMODE, NRcb		
100	4180.20	2967.55	1890.59	19666.20	12626.84	8199.64	7678.39	10914.14	∞	35036.58	51579.14	∞
1000	7695.49	3656.28	5170.80	35777.54	16521.14	23786.25	7705.26	10656.54	5236.20	36155.84	50456.44	24145.94
10000	4667.33	5068.59	5391.79	21445.34	22983.03	24824.15	5320.12	6396.72	6474.33	24634.90	29874.80	30162.13

N	NetMODE, GCf			NetMODE, GCc			NetMODE, GCfb			NetMODE, GCcb		
100	−0.74	−0.27	−0.16	0.04	0.39	0.60	0.50	0.77	0.90	1.14	1.42	1.71
1000	0.57	0.49	0.69	1.09	0.94	1.25	0.67	1.38	0.79	1.04	2.32	1.51
10000	0.80	0.70	0.76	1.23	1.14	1.04	0.76	0.86	0.83	1.18	1.29	1.29

N	NetMODE, LCf			NetMODE, LCc			NetMODE, LCfb			NetMODE, LCcb		
100	−0.98	−1.06	−1.02	0.24	−0.05	0.15	−0.83	−1.32	−1.09	0.25	−0.00	−0.04
1000	−0.95	−0.99	−1.05	0.23	0.16	0.17	−1.01	−0.99	−0.89	0.14	0.16	0.34
10000	−0.98	−1.00	−0.98	0.14	0.15	0.17	−0.99	−0.99	−0.99	0.15	0.16	0.15

N	NetMODE, ULCf			NetMODE, ULCc		
100	−0.80	−1.01	−0.80	0.28	0.27	0.21
1000	−1.01	−0.97	−1.01	0.17	0.15	0.15
10000	−0.99	−0.98	−0.98	0.16	0.16	0.17

Figure 6: Z-scores returned by Kavosh, FanMod, and NetMODE under various settings; E. Coli, 4-node subgraph census.

graphID	FanMod LC	NetMODE LC	NetMODE ULC
4382	−6.2336	−5.42	−5.34
4698	−39.906	−38.89	−38.32
4958	5405.2	230.59	233.21
13260	94.678	140.69	136.13
13278	∞	3178.05	3051.31
31710	∞	∞	∞

Figure 7: Concentration Z-scores returned by FanMod and NetMODE; power network (undirected), 4-node subgraph census.
